# An Assessment of the Expected Cost-Effectiveness of Quadrivalent Influenza Vaccines in Ontario, Canada Using a Static Model

**DOI:** 10.1371/journal.pone.0133606

**Published:** 2015-07-29

**Authors:** Ayman Chit, Julie Roiz, Samuel Aballea

**Affiliations:** 1 Health Outcomes and Economics—North America, Sanofi Pasteur, Toronto, Ontario, Canada; 2 Faculty of Pharmacy, University of Toronto, Toronto, Ontario, Canada; 3 Creativ-Ceutical Ltd, London, United Kingdom; 4 Creativ-Ceutical SARL, Paris, France; Centers for Disease Control, TAIWAN

## Abstract

Ontario, Canada, immunizes against influenza using a trivalent inactivated influenza vaccine (IIV3) under a Universal Influenza Immunization Program (UIIP). The UIIP offers IIV3 free-of-charge to all Ontarians over 6 months of age. A newly approved quadrivalent inactivated influenza vaccine (IIV4) offers wider protection against influenza B disease. We explored the expected cost-utility and budget impact of replacing IIV3 with IIV4, within the context of Ontario’s UIIP, using a probabilistic and static cost-utility model. Wherever possible, epidemiological and cost data were obtained from Ontario sources. Canadian or U.S. sources were used when Ontario data were not available. Vaccine efficacy for IIV3 was obtained from the literature. IIV4 efficacy was derived from meta-analysis of strain-specific vaccine efficacy. Conservatively, herd protection was not considered. In the base case, we used IIV3 and IIV4 prices of $5.5/dose and $7/dose, respectively. We conducted a sensitivity analysis on the price of IIV4, as well as standard univariate and multivariate statistical uncertainty analyses. Over a typical influenza season, relative to IIV3, IIV4 is expected to avert an additional 2,516 influenza cases, 1,683 influenza-associated medical visits, 27 influenza-associated hospitalizations, and 5 influenza-associated deaths. From a societal perspective, IIV4 would generate 76 more Quality Adjusted Life Years (QALYs) and a net societal budget impact of $4,784,112. The incremental cost effectiveness ratio for this comparison was $63,773/QALY. IIV4 remains cost-effective up to a 53% price premium over IIV3. A probabilistic sensitivity analysis showed that IIV4 was cost-effective with a probability of 65% for a threshold of $100,000/QALY gained. IIV4 is expected to achieve reductions in influenza-related morbidity and mortality compared to IIV3. Despite not accounting for herd protection, IIV4 is still expected to be a cost-effective alternative to IIV3 up to a price premium of 53%. Our conclusions were robust in the face of sensitivity analyses.

## Introduction

In 2000, the province of Ontario, Canada established a Universal Influenza Immunization Program (UIIP) to provide free influenza vaccine to all eligible members of the public. Trivalent inactivated influenza vaccine (IIV3) is the current vaccine used in Ontario. A new quadrivalent inactivated influenza vaccine (IIV4) has been recently licensed in Canada [1,2], and Ontario is faced with a decision on the adoption of IIV4 into its UIIP program.

Traditional IIV3 contains antigens from three viral strains: A(H1N1), A(H3N2), and one of two co-circulating B lineages, B(Victoria) or B(Yamagata). Each year, the World Health Organization (WHO) decides which viral strains should be included in the next seasonal influenza vaccine. However, accurately predicting which B-lineage strain will predominate in the upcoming season has proven to be a challenging task resulting in frequent mismatches with the vaccine strain [3]. During mismatch seasons, efficacy and effectiveness against the opposite B lineage were lower [4–10]. To address the issue of co-circulating B lineages, several manufacturers have developed IIV4s containing a strain from each B-lineage.

DiazGranados and colleagues [11], as well as Tricco and colleagues [12], showed that IIV3 provides cross-protection during influenza B mismatch situations. IIV3 efficacy was observed to be 86% against the same lineage and 51% against the opposite lineage influenza B [11]. Several studies have explored the expected public health and economic impact of IIV4 compared to IIV3 in the United States [13–15], United Kingdom [16], and Hong Kong [17]. Some studies included the emerging evidence on cross-protection [15,17], while others assumed no cross-protective benefit of IIV3 [13,14,16]. All studies, however, concluded that IIV4 can offer a health and economic benefit over IIV3 even if IIV4 was priced at a premium.

The objective of this analysis was to leverage the new emerging data on cross-protection and use a conservative static mathematical model to estimate the cost-utility and budget impact of replacing IIV3 with IIV4 within the context of Ontario’s UIIP.

## Methods

### Model Structure

Our modeling strategy was based on simulating the impact of IIV3 or IIV4 under the UIIP in an influenza season with an average influenza disease rate for the period between 2000–2001 and 2007–2008. We ran three basic simulations for this season in Ontario: 1) No influenza immunization program, 2) an IIV3 UIIP, and 3) an IIV4 UIIP. The main outputs of each simulation were the numbers of work days lost, general practitioner (GP) visits, emergency department (ED) visits, hospitalizations, and deaths. The model accounts for quality-adjusted life-years (QALYs) lost due to clinically relevant cases of influenza (cases resulting in a visit to the GP or ED, hospitalized cases, as well as influenza-related deaths).

Our model starts by calculating the rates of study outcomes in an unvaccinated population of Ontarians for the period between 2000–2001 and 2007–2008. No herd effects were considered during this calculation. The calculation was performed through the following equation:

$$R\prime_{ij}=\frac{R_{ij}}{C_{j}\left(1-VE_{3ij}\right)+\left(1-C_{j}\right)}$$

Where R’_ij_ is the mean rate of outcome i in age group j in a unvaccinated population over the study period; and where i considered 4 different outcomes: GP visits, ED visits, hospitalizations, and deaths, while j was stratified into 5 groups: ≤4, 5–19, 20–49, 50–64, ≥65 years of age. R_ij_ represents the mean rate of outcome i in age group j, over the study period when IIV3 was used under the UIIP in Ontario. C_j_ is the IIV3 coverage level over that same period. VE_3ij_ describes the IIV3 effectiveness against outcome i in age group j.

The rates of disease outcomes under the IIV3 program for the study period were available from the literature and defined above as R_ij_. However, for the IIV4 program we used the following equation to estimate the expected outcome rates:

$$R{\prime \prime }_{ij}=R\prime_{ij}\left(1-C_{ij}\right)+R\prime_{ij}C_{ij}\left(1-VE_{4ij}\right)$$

Where i and j still represent outcome type and age group respectively. R”_ij_ represents the outcome rate under the IIV4 program and VE_4ij_ represents the IIV4 effectiveness.

As we did not have direct estimates of VE_4ij_, we derived them from other data sources. First, IIV3 effectiveness in children and adults was obtained from Reed et al [13]. In addition, we used a recently published meta-analysis by DiazGranados et al [11] on the efficacy of inactivated vaccines against matched and mismatched circulating influenza B to account for cross-protection offered by IIV3 against mismatched circulating virus. Below is a description of our methodology.

We start by re-rewriting how Reed and colleagues defined IIV3 VE:

$$VE_{R3}=\sum_{i=I}^{IV}\left(x_{i}VE_{i}\right)$$

*VE*_*R3*_ is the vaccine effectiveness estimate from Reed et al for IIV3. *i* represents the various influenza strains where *I–IV* represent H1N1, H3N2, B _match_, B _mismatch_ respectively, *x*_*i*_ is the proportion of strain *i* relative to overall influenza circulation, and *VE*_*i*_ is the vaccine effectiveness against strain *i*.

Since Reed et al estimated that IIV3 provides no protection against B _mismatch_, we next calculated an adjusted VE for children and adults, *VE*_*CA3*_, that accounts for cross-protection where VE_B mismatch_ = 0.6(VE_B match_).

$$VE_{CA3}=\sum_{i=I}^{IV}\left(x_{i}VE_{i}\right)$$

Efficacy of IIV4 is derived similarly, assuming VE_B mismatch_ = VE_B match_:

$$VE_{CA4}=\sum_{i=I}^{III}\left(x_{i}VE_{i}\right)$$

i still represents the number of influenza strains, however, the VE against mismatched B is not considered. Instead, I–III represent H1N1, H3N2, and B respectively.

Beyer and colleagues [18] estimated that the VE in seniors was lower than that reported by Reed et al [13] for the overall population. This is in line with immunosenescence in seniors. To account for this, we calculated a correction factor, α, to reflect the difference between expected VE in the general population and that in seniors.

$$\alpha =\frac{VE_{Beyer}}{VE_{R3}}$$

Where, VE_Beyer_ is the vaccine effectiveness estimate from the Beyer et al meta-analysis. VE of IIV3 in seniors, VE_S3_, was then modeled in accordance with the equation below:

$$VE_{S3}=\alpha VE_{CA3}$$

Next, we modeled the VE of IIV4 in seniors, VE_S4_, in accordance with the equation below:

$$VE_{S4}=\alpha VE_{CA4}$$

After estimating the outcomes expected under each vaccine program, Quality of Life (QoL) data were combined with the outcome data to estimate the total number of QALYs a program would produce. Each outcome reduced the overall QoL as a function of the outcome duration and the disutility associated with influenza applied over that period. For influenza associated deaths, QALYs were lost from time of death until the life expectancy. The costs of the various outcomes were also calculated by multiplying outcome by their unit cost estimates. Finally, differences in total costs and total QALYs were computed and incremental cost effectiveness ratios (ICERs). The ICER was defined as (Cost_IIV4_ –Cost_IIV3_)/(Outcomes_IIV4_ –Outcomes_IIV3_).

Potential adverse effects of influenza vaccination were not represented in the model. These generally involve mild-to-moderate injection-site or systemic effects that are transient with negligible cost and little impact on quality of life [19]. Further, studies have shown no significant differences between the adverse event profile of IIV3 and IIV4 [19].

S1 Model contains a copy of the functioning model in Microsoft Excel (S1 Model).

### Model Inputs

Table 1 summarizes all the input parameters of the model. Results from two publications were used extensively. Firstly, Kwong et al provided rates of coverage, GP consultation, ED visit, hospitalization, and death by age group [20]. Secondly, we relied on Sander et al’s work for economic data including QALYs lost due to influenza and Ontario-based cost data [21].

**Table 1:** Model inputs.

Input	Mean	DSA* Range	PSA^†^ distribution	Source
**Population characteristics (Ontario)**				
**Population size**				
≤4 years	704,260	NA	NA	[34]
5–19 years	2,340,140	NA	NA	[34]
20–49 years	5,292,680	NA	NA	[34]
50–64 years	2,636,405	NA	NA	[34]
≥65 years	1,878,330	NA	NA	[34]
**Life expectancy (years)**				
≤4 years	79.56	NA	NA	[34]
5–19 years	69.40	NA	NA	[34]
20–49 years	47.23	NA	NA	[34]
50–64 years	27.23	NA	NA	[34]
≥65 years	12.96	NA	NA	[34]
**Vaccine coverage**				
≤4 years	31.00%	0.2514–0.3686	Normal (0.31;0.01)	[20]
5–19 years	31.00%	0.2514–0.3686	Normal (0.31;0.01)	[20]
20–49 years	27.00%	0.2107–0.3293	Normal (0.27;0.01)	[20]
50–64 years	47.00%	0.3971–0.5429	Normal (0.47;0.01)	[20]
65–74 years	71.00%	0.6566–0.7634	Normal (0.71;0.01)	[20]
75–84 years	81.00%	0.7655–0.8545	Normal (0.81;0.01)	[20]
≥85 years	78.00%	0.7655–0.7945	Normal (0.78;0.02)	[20]
**Influenza-related GP** ^**‡**^ **consultation rate (per 100,000)**				
≤4 years	1,932.81	1,917–1,949	LogNormal (1,933;68)	[20,23,24]
5–19 years	1,441.87	1,430–1,454	LogNormal (1,442;36)	[20, 23,24]
20–49 years	636.75	629–645	LogNormal (637;16)	[20, 23,24]
50–64 years	457.90	450–466	LogNormal (458;17)	[20, 23,24]
65–74 years	634.13	624–645	LogNormal (634;29)	[20, 23,24]
75–84 years	1,287.67	1,273–1,303	LogNormal (1,288;59)	[20, 23,24]
≥85 years	3,224.15	3,200–3,248	LogNormal (3,224;149)	[20, 23,24]
**Influenza-related ED** ^**§**^ **consultation rate (per 100,000)**				
≤4 years	556.88	548–566	LogNormal (557;20)	[20, 23,24]
5–19 years	180.17	176–184	LogNormal (180;5)	[20, 23,24]
20–49 years	84.87	82–88	LogNormal (85;2)	[20, 23,24]
50–64 years	63.28	60–66	LogNormal (63;2)	[20, 23,24]
65–74 years	181.28	176–187	LogNormal (181;8)	[20, 23,24]
75–84 years	398.31	390–406	LogNormal (398;18)	[20, 23,24]
≥85 years	1148.01	1,134–1,162	LogNormal (1,148;53)	[20, 23,24]
**Influenza-related hospitalization rate (per 100,000)**				
≤4 years	54.14	51.3–56.8	LogNormal (54;2)	[20, 23,24]
5–19 years	2.98	2.4–3.7	LogNormal (3;0)	[20, 23,24]
20–49 years	2.75	2.4–3.7	LogNormal (3;0)	[20, 23,24]
50–64 years	4.59	4.2–5.9	LogNormal (5;0)	[20, 23,24]
65–74 years	34.87	32.3–37.9	LogNormal (35;2)	[20, 23,24]
75–84 years	131.22	126.3–135.9	LogNormal (131;6)	[20, 23,24]
≥85 years	432.44	423.3–440.8	LogNormal (432;20)	[20, 23,24]
**Influenza-related death rate (per 100,000)**				
≤4 years	0.40	0.24–0.63	LogNormal (0.4;0.01)	[20, 23,24]
5–19 years	0.40	0.24–0.63	LogNormal (0.4;0.01)	[20, 23,24]
20–49 years	0.40	0.19–0.74	LogNormal (0.4;0.02)	[20, 23,24]
50–64 years	1.30	0.89–1.84	LogNormal (1.3;0.06)	[20, 23,24]
65–74 years	6.20	5.15–7.40	LogNormal (6.2;0.33)	[20, 23,24]
75–84 years	28.40	26.06–30.89	LogNormal (28.4;1.52)	[20, 23,24]
≥85 years	134.30	129.13–139.62	LogNormal (134.3;7.16)	[20, 23,24]
**Number of non-consulting cases for one consulting**				
All ages	0.49	0.2–1		[20]
**Quality of life**				
**Population utility norms**				
≤4 years	0.92	0.911–0.929	Normal (0.92;0.0047)	[35,36]
5–19 years	0.89	0.867–0.915	Normal (0.89;0.0122)	[35,36]
20–49 years	0.90	0.846–0.957	Normal (0.9;0.0281)	[35,36]
50–64 years	0.85	0.787–0.907	Normal (0.85;0.0306)	[35,36]
≥65 years	0.80	0.670–0.929	Normal (0.8;0.0661)	[35,36]
**QALYs** ** **lost to influenza**				
≤4 years	0.0146	0.0065–0.0146	Beta (8.4;569)	[21]
5–19 years	0.0146	0.0065–0.0146	Beta (8.4;569)	[21]
20–49 years	0.0174	0.0097–0.0245	Beta (14.6;826)	[21]
50–64 years	0.0174	0.0044–0.0245	Beta (3.6;206)	[21]
≥65 years	0.0293	0.0233–0.0349	Beta (79.8;2,642)	[21]
**Workdays lost**				
**Total annual hours lost to influenza (%)**				
≤4 years	0	NA	NA	[37]
5–19 years	0.0888%	NA	NA	[37]
20–49 years	0.0993%	NA	NA	[37]
50–64 years	0.0451%	NA	NA	[37]
≥65 years	0.0451%	NA	NA	[37]
**Monetary costs ($CAD)**				
GP consultation^**1**^	35	18–60	LogNormal (35;13)	[21]
ED consultation^**1**^	220	183–371	LogNormal (220;77)	[21]
Hospitalization^**1**^	6,418	2,075–21,548	LogNormal (6,418;7,720)	[21]
Medication	3	NA	Uniform (1;5)	[38]
Vaccine (per dose)				
IIV3^**††**^	5.5	NA	NA	Sanofi Pasteur
IIV4^**‡‡**^	7	NA	NA	Sanofi Pasteur
Hourly labor	24.46	NA	NA	[26]
**Strain-specific vaccine efficacy**				
A/H1N1	0.58	0.457–0.698	Beta (36.57;26.48)	[13]
A/H3N2	0.53	0.410–0.648	Beta (34.91;30.96)	[13]
Matched B	0.47	0.267–0.678	Beta (9.93;11.2)	[13]
Mismatched B	0.28	0.166–0.411	Beta (13.98;35.96)	[11;13]
**Discount rate (%)**	5	3–7		[39]

^1^ Inflated to 2012 using Statistics Canada, CANSIM, table 326–0021, "Health and Personal care > Health care > Health care services".

*DSA: Deterministic Sensitivity Analysis

^†^PSA: Probabilistic Sensitivity Analysis

^‡^GP: General Practitioner

^§^ED: Emergency Department

^**^QALY: Quality-adjusted life-years

^††^IIV3: Trivalent Inactivated Influenza Vaccine

^‡‡^IIV4: Quadrivalent Inactivated Influenza Vaccine

Other data were obtained from a search in PubMed and statistics data bases. We used Canadian and Ontarian statistics whenever possible. In instances where Canadian statistics were not available, we referenced data from other countries with a geographic and socio-economic milieu similar to Ontario and Canada, such as the United States. All cost data were inflated to 2012 Canadian dollars using the medical component of the consumer price index for Canada [22]. Beyond that all outcomes and cost were discounted at a 5% annual rate.

### Epidemiological Inputs

The study by Kwong et al provided annual influenza-associated outcome rates (GP visit, ED visit, hospitalization, death) from the 2000–2001 season to the 2003–2004 season [20]. Given the annual variability in the epidemiology of influenza, we were concerned that this limited time period would not constitute a representative sample to provide a valid estimate of the average burden of influenza in Ontario under the UIIP. For instance, it is possible that during these four years influenza circulation would be high and dominated by Influenza B. This would clearly bias our analysis in favor of IIV4. Accordingly, we leveraged US data on influenza-related outcome rates to employ an extrapolation beyond the Kwong et al data. This extrapolation involved two steps. First, we used data from the US to estimate the ratio between the influenza-related events in US [23,24] and Ontario [20] during the influenza seasons 2000–2001 to 2003–2004. Secondly, we used this ratio to adjust US-based influenza outcome rates for the seasons 2004–2005 to 2007–2008. The adjusted rates for the aforementioned period were then taken to be representative of Ontario’s outcome rates during the same time period.

Data on US influenza-related hospitalization rates for the period 2000–2001 to 2007–2008 were obtained from the work of Zhou et al [24]. The data from this source were stratified by age and by influenza subtype (A/H3N2, A/H1N1, B/Yamagata, and B/Victoria). Data on US influenza-related death rates for the seasons 2000–2001 to 2006–2007 were published in the US Centers for Disease Control’s 2010 Mortality and Morbidity Weekly Report [23]. These data were only stratified by age, and we used data from Reed et al. to stratify by strain [13]. Since the data in the Kwong et al study were not stratified by influenza subtype, we superimposed the stratification from the aforementioned US data sources for the period 2000–2001 to 2003–2004 onto the Kwong data.

### Cost Inputs

Costs were estimated from the perspective of the Ontario ministry of health (MOH) and the provinces as a whole (societal perspective). The health care system perspective included the cost of administering vaccine and the cost of managing influenza disease as it is covered by the health care system in Ontario. The societal perspective was inclusive of the health care system perspective and further considers medication costs for Ontarians below the age of 65, as well as productivity losses for the working population in Ontario.

IIV3 and IIV4 are purchased under a Canadian federal tender where it is possible for different manufacturers to have different prices. The average prices paid by Ontario for IIV3 and IIV4 are not publically available. Further, as tenders are renewed either every three years or annually, the prices will change over time. In this analysis we used an IIV3 price of $5.5/dose and an IIV4 price of $7/dose in the base case. We then conducted a sensitivity analysis on the difference in price between the two vaccines (IIV4 price premium).

Productivity losses were estimated based on the friction method to include losses due to absenteeism and losses due to premature death [25]. Work loss was assumed to occur amongst employed Ontarians experiencing symptomatic influenza, an influenza-associated hospitalization, or an influenza-related death (limited to 90 days post-death). The cost to a firm due to the absence of an employee depends on several factors. If the firm must replace the employee with an overtime/temporary worker, then the cost to the firm is equal to the overtime/temporary worker wages. If the employee can’t be replaced through an overtime/temporary worker, a firm might be able to sustain some degree of production while the employee is off sick. In the case that full production is sustained while the employee is off sick; then there is no cost to the firm as a result of the illness. In contrast, if the firm loses all the production associated with that employee not being at work, then the cost to the firm is the wages it pays the employee while they are at home, plus the opportunity cost, i.e. the profitability the employee would have generated for the firm. As data defining many of these parameters are absent, we simplified by assuming that the firm’s cost due to the short term illness of one of its employees is equal to the average wages paid to that employee in Canada while they are off work sick. We note that there are two estimates of the hourly labor costs in Canada: $24.46/hour reported by Statistics Canada [26], and $46.4/hour reported by the Organization for Economic Co-operation and Development (OECD) [27]. Conservatively, we used the hourly labor cost reported by Statistics Canada in our base case analysis.

### Sensitivity Analysis

Given the uncertainty around the difference in prices between IIV3 and IIV4 in Canada, we conducted a sensitivity analysis of the impact on the size of IIV4 price premium on the cost-effectiveness of the vaccine. Productivity costs also pose a source of non-statistical uncertainty and can be influential on the cost-effectiveness results. As such, we conducted a scenario analysis using $37/hour which is a 50% premium over the base case labor cost and also present a one-way sensitivity analysis of the impact of hourly Canadian worker wage on the cost-effectiveness of the IIV4. Further, we conducted one-way deterministic sensitivity analyses on the statistical distributions of all input parameters and results were presented as a tornado diagram. A multivariate probabilistic sensitivity analysis (PSA) of parameter statistical uncertainty was also conducted. PSA results were presented as a cost-effectiveness acceptability curve (CEAC).

## Results

The use of IIV4 instead of IIV3 in an average Ontario influenza season would save 12,329 work days and avoid 1,380 GP visits, 303 ED visits, 27 hospitalizations, and 5 deaths. The distribution of outcomes avoided by age is shown in Table 2. Most GP consultations would be avoided in the 5–19 and 20–49 years-old age groups, partly because of the larger size of these specific age groups. Most hospitalizations and almost all deaths would be prevented in individuals 50 years of age or older. The higher vaccination costs of IIV4 would be partially offset by reductions in consultation and hospitalization costs as well as gains in productivity. The net annual budget impacts would be $4.8 million from a societal perspective and $7.2 million from a MOH perspective. Table 3 summarizes the cost offsets associated with the use of IIV4.

**Table 2:** Health outcomes avoided with IIV4 in Ontario by age group.

Age group	GP* consultations	ED^†^ visits	Hospitalizations	Deaths	Life-years gained	QALYs^‡^ gained	Workdays saved
≤4y	115.7	33.3	2.8	0.0	0.5	2.36	-
5–19y	286.9	35.8	0.6	0.1	1.5	6.1	490
20–49y	243.7	32.5	1.1	0.1	2.5	7.0	8,131
50–64y	172.1	23.8	1.6	0.4	6.1	8.4	3,014
≥65y	562.0	177.4	20.9	4.7	38.1	52.1	694
**Total**	1,380	303	27	5	49	76	12,329

*GP: General Practitioner

^†^ED: Emergency Department

^‡^QALY: Quality-adjusted life-years

**Table 3:** Cost offsets with IIV4 in Ontario ($CAD) by age group.

Age group	GP* consultations	ED† visits	Hospitalizations	Medications	Productivity losses due to illness	Productivity losses due to death
≤4y	$ 4,939	$ 8,945	$ 21,994	$ 668	-	-
5–19y	$ 12,243	$ 9,616	$ 4,664	$ 1,447	$ 38,688	$ 433
20–49y	$ 10,401	$ 8,714	$ 8,282	$ 1,238	$ 1,581,318	$ 1,842
50–64y	$ 7,345	$ 6,381	$ 12,159	$ 878	$ 596,161	$ 5,981
≥65y	$ 23,988	$ 47,595	$ 163,482	$ 3,316	$ 113,136	$ 54,852
**Total**	$ 58,916	$ 81,251	$ 210,581	$ 7,548	$ 2,329,303	$ 63,107

*GP: General Practitioner

^†^ED: Emergency Department

The incremental cost-effectiveness ratios (ICERs) for switching from IIV3 to IIV4 are shown by age group and perspective in Table 4. For the base case, we estimated ICERs of $63,773/QALY from a societal perspective and $94,248/QALY from a MOH perspective. IIV4 was most cost-effective in individuals 65 years of age or older. The ICERs for this age group were $33,870/QALY from a societal perspective and $36,034/QALY from a MOH perspective.

**Table 4:** Incremental cost-effectiveness ratios of IIV4 versus IIV3 by age group.

Age group	Incremental cost-effectiveness ratios ($CAD/QALY^†^)	
	MOH* perspective	Societal perspective
All ages	$ 94, 248	$ 62,792
≤4y	$ 112,274	$ 112,017
5–19y	$ 174,525	$ 167,856
20–49y	$ 303,851	76,351
50–64y	$ 217,878	$ 146,192
65+	$ 36,034	$ 32,864

*MOH: Ministry of Health

^†^QALY: Quality-adjusted Life-years

**Fig 1:**
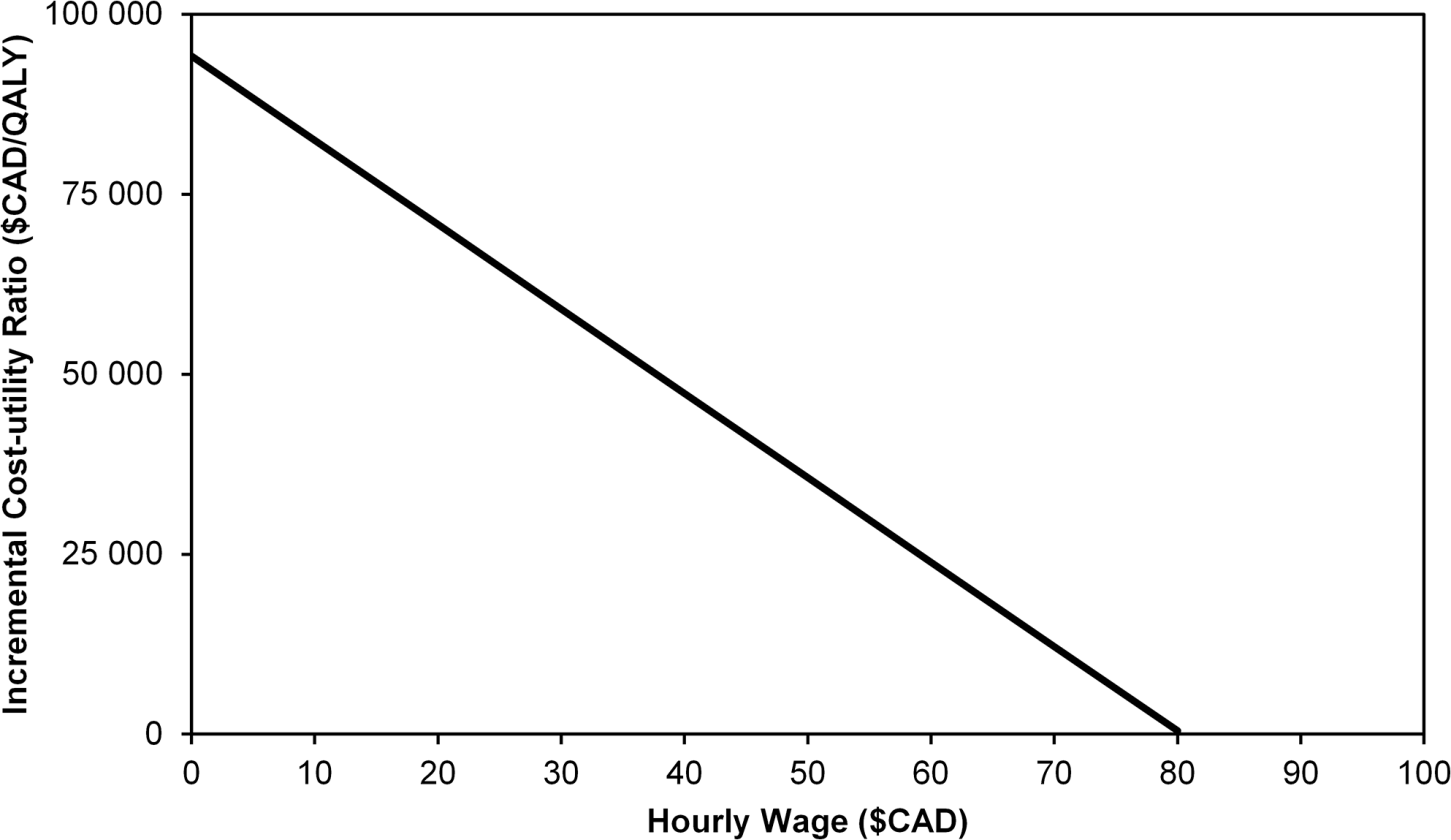
One-way sensitivity analyses (hourly wage).

**Fig 2:**
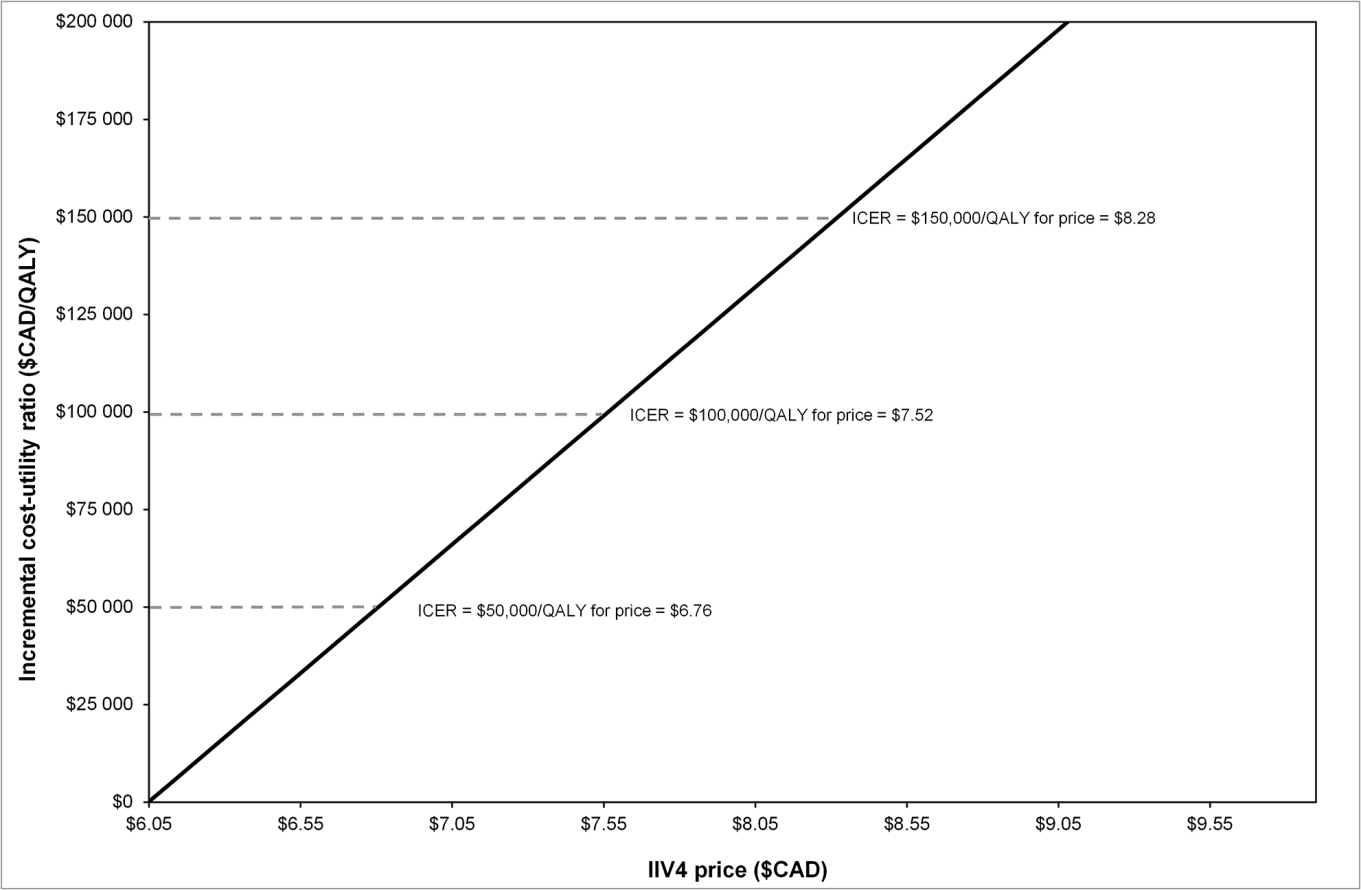
Threshold analysis: Incremental cost-effectiveness ratio (ICER) versus price of IIV4.

The cost faced by firms due to influenza-related absenteeism is an influential parameter in the cost-effectiveness analysis. In Fig 1, we present a 1-way sensitivity analysis for the impact of the hourly labor cost on the ICER. Source data did not contain any statistical uncertainty estimates for the hourly labor cost; however, we did find two different estimates for the mean hourly labor costs in Canada. The figures reported by Statistics Canada were presented in the base case. In a scenario analysis, we used the hourly wage of $37/hour and found that this reduced the base case ICER to $47,489/QALY. Fig 2 shows how the ICER from the societal perspective varied as a function of the IIV4 price premium. IIV4 would be cost-neutral (more effective at the same cost) at a price of $6.10/dose, and the ICER would be $150,000/QALY at a price of $8.40/dose. Accordingly, IIV4 is dominant at a price premium of 11% and remains below $150,000/QALY–(the likely upper limit of a Canadian cost effectiveness threshold) up to a price premium of 53%.

**Fig 3:**
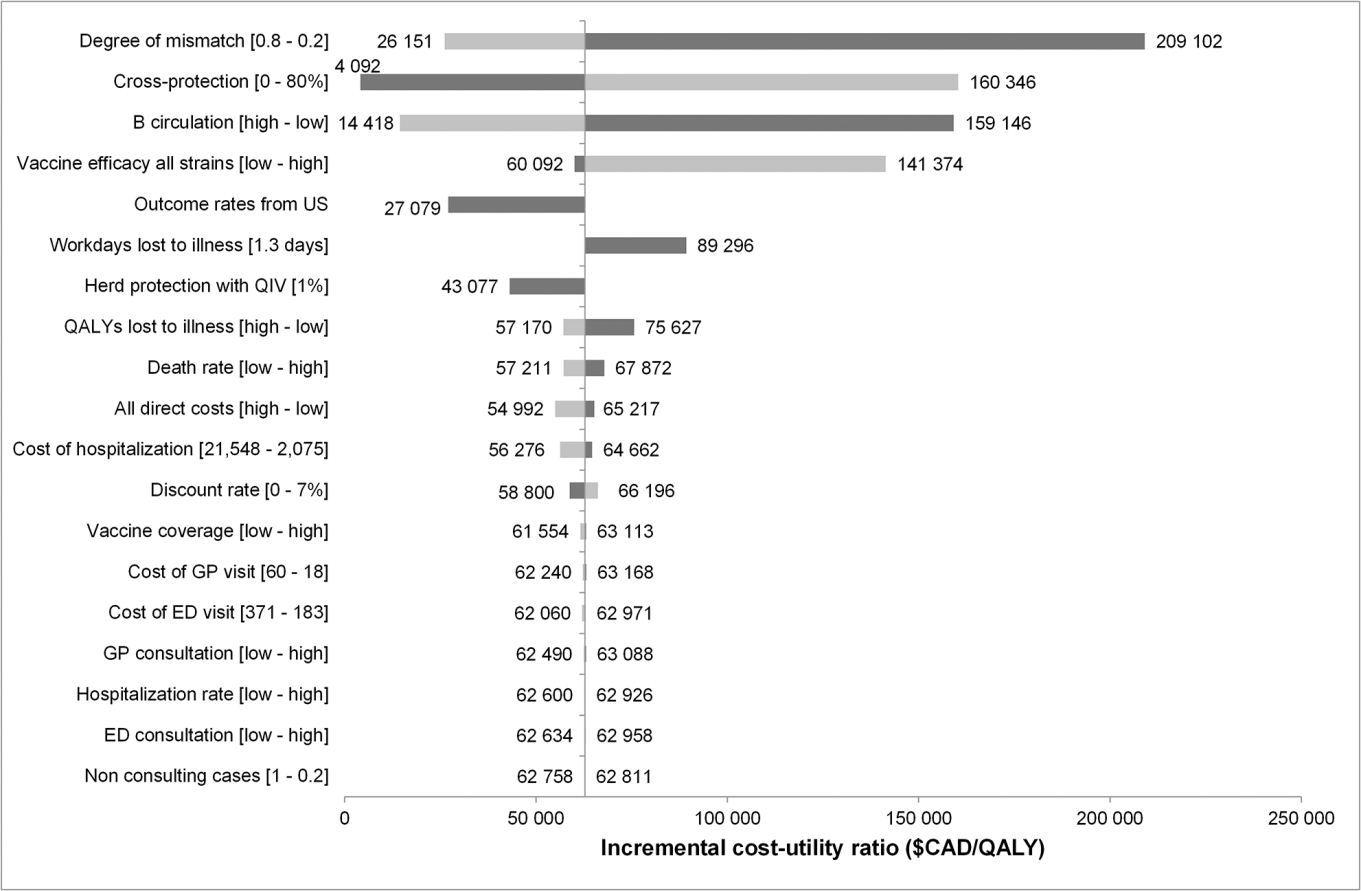
Deterministic sensitivity analysis (societal perspective).

**Fig 4:**
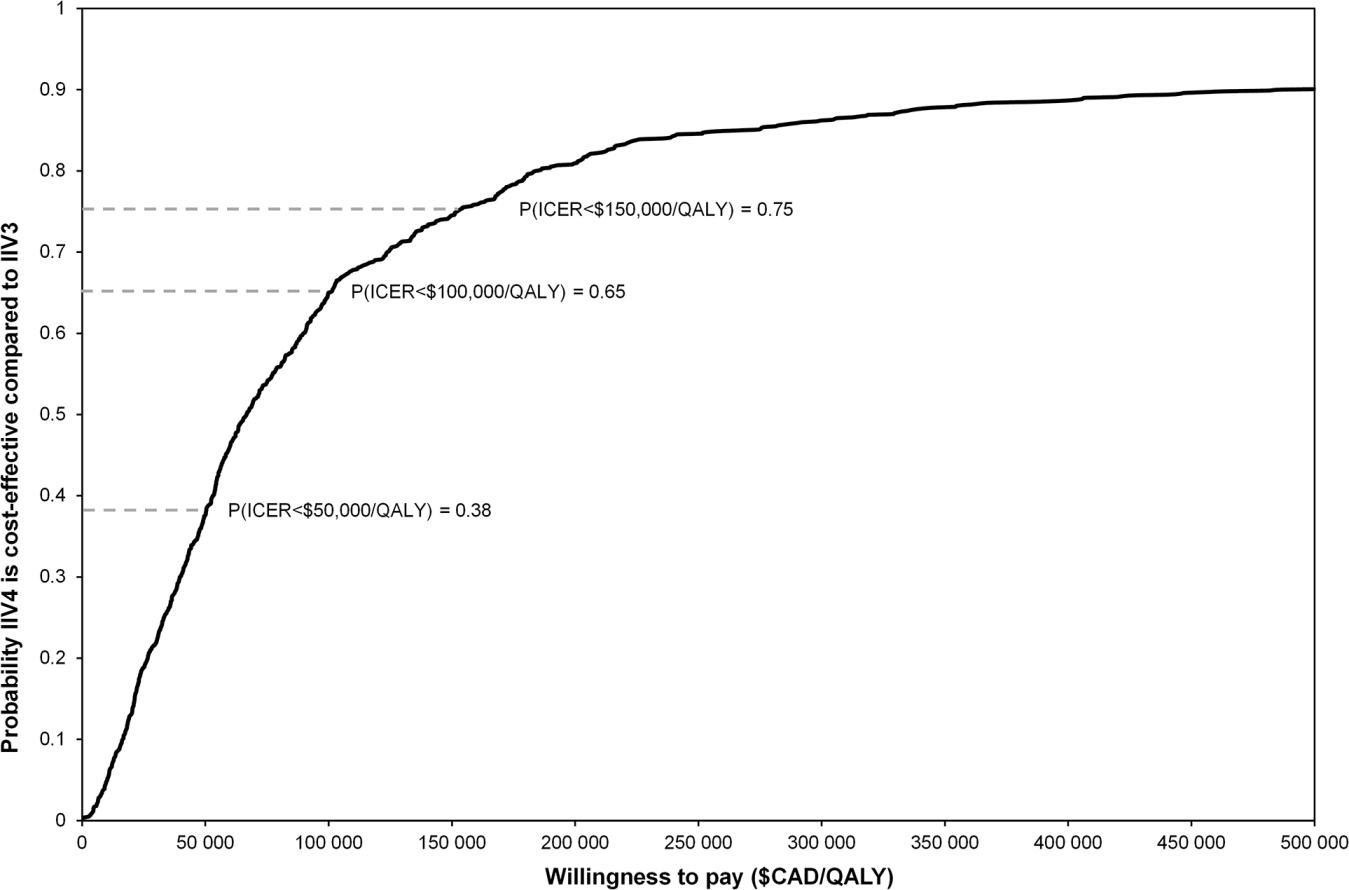
Cost-effectiveness acceptability curve.

A tornado diagram highlighting the impact of univariate statistical uncertainty on the cost-effectiveness estimates is shown in Fig 3. The model is most sensitive to the degree of mismatch, cross-protection, and circulation of B strain. The PSA results are presented in Fig 4 where we find that 65% of the PSA simulations provided results below $100,000/QALY.

## Discussion

The findings of this static modeling study show that IIV4 is expected to reduce the burden of influenza compared to IIV3 by preventing disease, reducing health care costs, and improving productivity in the work place. From a societal perspective, IIV4 would be cost-effective for Ontario’s UIIP at a price below $8.40/dose (53% price premium over IIV3). If the price of IIV4 drops below $6.10/dose (11% price premium over IIV3) an IIV4-based UIIP will be more effective and less costly than an IIV3 based UIIP.

The International Society of Pharmacoeconomics and Outcomes Research and the Society of Medical Decision Making published a joint position paper on modeling standards for estimating the expected epidemiological impact and cost-effectiveness of immunization programs [28]. The recommendations indicate that dynamic models are necessary tools for investigating the impact of an intervention that affects disease transmission. Static models are only appropriate when their expectations suggest that the intervention would be cost effective. This is because dynamic models incorporating herd immunity would most likely only show additional benefits in favor of the intervention. It has been demonstrated by Loeb and colleagues, in a randomized controlled trial, that IIV3 can induce indirect protection though herd effects [29]. Accordingly, our estimation of the cost-effectiveness of IIV4 in Ontario is conservative and would support a decision to switch to IIV4 at prices below $8.40/dose. Further, our model would not be a useful tool to explore the cost-effectiveness of IIV4 at prices higher than $8.40/dose, since this price approaches the likely upper limit of the Canadian cost-effectiveness threshold.

It should be noted that Canada does not have an explicit cost-effectiveness threshold used to inform adoption decisions. Further, the mechanism of how economic information factor into vaccine adoption decisions is heterogeneous across the provinces and not transparent [30]. To estimate the upper bound of the implicit threshold within Canadian healthcare we reviewed the ICERs of the 10 most recently approved and funded oncology medicines in Canada and found that 150,000/QALY was the mean ICER [31]. We selected Oncology medicines as they are known to be less cost-effective than other medical interventions, to the extent that in the UK there is an explicitly higher threshold allowed for end of life treatments.

Our model has a novel feature in that it considers data from DiazGranados et al [11] and Tricco et al [12]. Both groups recently demonstrated that IIV3 provides cross-protection during influenza B mismatch situations. We were able to incorporate the estimate that IIV3 provides 60% of the matched efficacy against lineage-mismatched influenza B [11]. It should be noted that these data were from studies of IIV3 administered to children and young adults; there were no data in individuals 65 years of age and older. We made the assumption that the size of this effect was constant across the age spectrum. In our deterministic sensitivity analysis, if the cross-protection was increased to 80% of the matched influenza B efficacy (across all age groups), the resulting base-case ICER would be $160,346/QALY.

The major limitation of our model is related to the quality of the Ontario influenza-related outcomes data. The only data available for Ontario were from a publication by Kwong and colleagues [20]. These data were limited in three major ways; they were not stratified by influenza subtype, they used outcome definitions that are not sufficiently sensitive to capture some influenza-related complications, and they were limited to only 4 years after the introduction of the UIIP. The limitations of the data are further highlighted through a contrast with the US where researchers have access to time-series data for influenza-related morbidity and mortality stratified by age and by influenza subtype [23,24].

In the model, we leveraged the rich US data to make extrapolations for the province of Ontario. In doing so, we noted that hospitalization and death rates in some age groups were from 2-fold to more than 10-fold lower in Canada compared to the US. For example, in adults 50–64 years of age, the Ontario rate of influenza-related hospitalization was 4.6/100,000 [20] while the reported US rate was 63.2/100,000 [23]. Some of this difference might be related to the underlying health care systems in the two countries. However, some of the difference may also be explained by differences in the outcome definitions used to approximate the burden in each country. To some extent, Kwong et al’s estimates of influenza-related hospitalizations underestimate the actual burden of disease in Ontario. This is primarily because the authors only considered influenza-related hospitalizations coded as respiratory admissions. It is well established that influenza is implicated in triggering major circulatory medical events [32]. As such, the definition used by Kwong et al is clearly not broad enough to capture influenza-related hospitalizations that are coded as circulatory admissions. US estimates reported by several authors consider both respiratory and circulatory influenza-related hospitalizations and deaths [24,33]. To remain conservative, we downward-adjusted the US outcome rates reported during our study period to make them consistent with those estimated by Kwong et al as described in the methods section. In one of our deterministic sensitivity analyses, we removed the downward adjustment imposed on the US outcome rates. In doing so, the ICER was reduced to $28,351/QALY. This point highlights a conservative feature of our economic model and the need for better and more accessible influenza-related outcome data in Ontario and Canada.

In conclusion, our findings from this economic evaluation indicate that a switch in Ontario’s UIIP from IIV3 to IIV4 would prevent influenza-related lost productivity, GP consultations, hospitalizations, and deaths. IIV4 remains cost-effective up to a 53% price premium over IIV3.

## Supporting Information

S1 Model IIV4 cost-effectiveness model in Microsoft Excel. (XLSM)
